# The association between time in the glucose target range and abnormal ankle-brachial index: a cross-sectional analysis

**DOI:** 10.1186/s12933-022-01718-y

**Published:** 2022-12-13

**Authors:** Yinghua Wei, Chunyan Liu, Yanyu Liu, Zhen Zhang, Zhouqin Feng, Xinyi Yang, Juan Liu, Haiyan Lei, Hui Zhou, Qiuyue Shen, Bin Lu, Ping Gu, Jiaqing Shao

**Affiliations:** 1 Department of Endocrinology, Affiliated Jinling Hospital, Medical School of Nanjing University, 305 East Zhongshan Road, Nanjing, 210002 Jiangsu China; 2 Department of Endocrinology, Affiliated Hospital of Jiangnan University, Wuxi, Jiangsu China; 3 Department of Endocrinology, Jinling Hospital, The First School of Clinical Medicine, Southern Medical University, Nanjing, China; 4 Department of Endocrinology, The affiliated Jinling Hospital of Nanjing Medical University, Nanjing, Jiangsu China

**Keywords:** Ankle-brachial index, Time in range, Continuous glucose monitoring, Hemoglobin A1C, Type 2 diabetes

## Abstract

**Background:**

Time in range (TIR), a novel proxy measure of glucose control, is found closely related to diabetic microangiopathy and some other chronic complications, but the correlation between TIR and lower limb angiopathy has not been studied yet. Our purpose is to explore the relationship between TIR and abnormal ankle-brachial index(ABI) in type 2 diabetes.

**Methods:**

We retrospectively collected patients’ information from the database and performed cross-sectional analysis. A total of 405 type 2 diabetes patients were enrolled in this study. ABI was measured and patients were stratified into low, normal, and high groups according to ≤ 0.9, > 0.9 and < 1.3, ≥ 1.3 ABI values. All patients underwent continuous glucose monitoring(CGM), and TIR was defined as the percentage of time in which glucose was in the range of 3.9–10 mmol/L during a 24-h period. Correlations between TIR and abnormal ABI were analyzed using Spearman analysis. And logistic regression was used to explore whether TIR is an independent risk factor for abnormal ABI.

**Results:**

The overall prevalence of abnormal ABI was 20.2% (low 4.9% and high 15.3%). TIR was lower in patients with abnormal ABI values (P = 0.009). The prevalence of abnormal ABI decreased with increasing quartiles of TIR (P = 0.026). Abnormal ABI was negatively correlated with TIR and positively correlated with hypertension, age, diabetes duration, UREA, Scr, ACR, TAR, MBG, and M values (P < 0.05). The logistic regression revealed a significant association between TIR and abnormal ABI, while HbA1C and blood glucose variability measures had no explicit correlation with abnormal ABI. Additionally, there was a significant difference in LDL between the low and high ABI groups (P = 0.009), and in Scr between normal and low groups (P = 0.007). And there were significant differences in TIR (P = 0.003), age (P = 0.023), UREA (P = 0.006), ACR (P = 0.004), TAR (P = 0.015), and MBG (P = 0.014) between normal and high ABI groups, and in diabetes duration between both normal and low (P = 0.023) and normal and high (P = 0.006) groups.

**Conclusions:**

In type 2 diabetes patients, abnormal ABI is associated with lower TIR, and the correlation is stronger than that with HbA1C. Therefore, the role of TIR should be emphasized in the evaluation of lower limb vascular diseases.

## Background

Diabetes, as a common chronic disease, has become an increasingly serious public health burden [1]. Its incidence is increasing year by year all over the world, and it’s estimated that in 2021, 537 million adults (20–79 years old) worldwide will have diabetes, and this number is expected to grow to 643 million by 2030 and 784 million by 2045 [2].

Cardiovascular death is the first major cause of premature death in patients with diabetes [3]. Ankle-brachial index(ABI) is the ratio of ankle systolic blood pressure to brachial artery systolic blood pressure, which was originally used in the non-invasive diagnosis of peripheral arterial disease (PAD). Because of its simplicity, non-invasive way, low cost, high repeatability, and strong specificity [4, 5], ABI has been widely used to screen for cardiovascular disease and has been reported to be an independent marker of cardiovascular morbidity and mortality. The American Heart Association recommends an ABI threshold of 0.90 for diagnosing PAD [6], and ABI ≤ 0.90 can replace other noninvasive tests in clinical practice as a simple and useful tool for determining severe stenosis in PAD [7] and is widely used as a marker for the presence and progression of PAD in major cardiovascular trials. High ABI values (> 1.3–1.4) are also common in diabetics and are considered an indicator of cardiovascular system damage and a marker of arterial calcification [8]. ABI measurements revealed a large number of asymptomatic patients with abnormal ABI in internal medicine inpatients, and an increase in cardiovascular mortality was observed in both low and high ABI patients, suggesting that patients admitted for any reason should be tested for ABI and initiate appropriate prevention [5]. The American Diabetes Association recommends measuring the ABI in all patients over the age of 50 with diabetes or any PAD symptoms or other cardiovascular risk factors [9].

Hemoglobin A1C(HbA1C) is a traditional index evaluating the average glucose level over the last 2–3 months. Previous studies have shown that HbA1C is positively correlated with Carotid artery intima-media thickness [10] and the progression of coronary artery calcification [11]. However, so far, in the studies of macrovascular complications using HbA1C as the primary endpoint, some have not observed expected significant benefits of glucose control on cardiovascular disease. It has previously been attributed to multiple risk factors such as blood pressure and blood lipids, as well as other so-called residual cardiovascular risks [12–14]. Hypoglycemia masked by HbA1C and glucose fluctuations that cannot be accurately reflected by HbA1C may also be important cardiovascular risks.

Time in range (TIR), especially measured by continuous glucose monitoring (CGM), is defined as the amount of time an individual spends within the target glucose range (3.9–10 mmol/L). Studies have confirmed that TIR has a stronger correlation with microangiopathy than HbA1C. Importantly, studies have confirmed that small improvements of 5–10% in TIR can translate into significant glycemic benefits [15], and prospective studies showed that every 10% increase in treatment-induced TIR was associated with an 18% decrease in proteinuria [16]. However, there is still a lack of research on the correlation between TIR and cardiovascular disease outcomes, especially PAD in diabetic patients. Whether TIR is more correlated with diabetic macrovascular complications than HbA1C, and whether the application of TIR as a target and endpoint may become an important strategy for controlling macroangiopathy still needs further research. Thus, the purpose of this study was to explore whether TIR is correlated with abnormal ABI in type 2 diabetes patients.

## Methods

### Participants

There were 460 diabetic patients that met the inclusion criteria in the analyzed period, 55 patients were eliminated from the analysis for lack of detailed information such as lipid profiles, albumin-to-creatinine ratio, fundus conditions, smoking history, and other relevant variables, or insufficient data of CGM. In this study, a total of 405 adult patients (age ≥ 18 years old) admitted to the Department of Endocrinology, Jinling Hospital, Nanjing University, from December 2017 to September 2020 were recruited. The inclusion criteria were type 2 diabetes diagnosed according to 1999 WHO diagnostic criteria. Patients with acute complications of diabetes; severe infection, trauma, surgery, and other acute stress states; severe liver, kidney, and other organ dysfunction; severe electrolyte disorder; a history of taking narcotic or psychotropic drugs; a recent history of alcohol poisoning were excluded. The study was approved by the ethics committee of Jinling Hospital affiliated with Nanjing University.

### Measurements

We obtained baseline characteristic information from the electronic medical record system retrospectively, including age, sex, diabetes duration, height, weight, systolic blood pressure, diastolic blood pressure, and diabetes complications. Body mass index (BMI) was calculated as weight (kg)/height (m) ^2^. Biochemical measurements included HbA1C, total cholesterol (TC), triglyceride (TG), high-density lipoprotein (HDL), low-density lipoprotein (LDL), C-reactive protein (CRP), blood UREA, serum creatinine (Scr), blood uric acid (UA), and urinary albumin/creatinine ratio (ACR) were detected. The diagnosis of hypertension was based on the criteria suggested in The Guidelines for Prevention and Treatment of Hypertension in China (Revised edition) 2021: systolic blood pressure ≥ 140 mmHg and/or diastolic blood pressure ≥ 90 mmHg. HbA1c was measured by high-performance liquid chromatography (HLC-723G8 Automatic Hemoglobin a1c analyzer, TOSOH, Japan). All the above biochemical indexes were detected under the condition of fasting for more than 10 h. Former or current smokers were considered to have a history of smoking. The diagnostic criteria for diabetic kidney disease was at least two tests with an ACR of more than 30 mg/g. Diabetic retinopathy was identified by the same ophthalmologist through fundus examination and stereo fundus photography after pupil dilation.

For CGM parameters, we used the continuous glucose monitoring system (Meiqi Company) for 72 h continuous blood glucose monitoring when patients were upon admission to the diabetes clinic and we calibrated the monitor by measuring capillary blood glucose at least 4 times per day as required by the instruction. During this process, intensive activities were prohibited. Based on the raw blood glucose data recorded by the system, indicators of mean blood glucose (MBG) and blood glucose variability (GV) were calculated using the EasyGV Version 9.0R2 software provided by Oxford University, Including standard deviation (SD), mean glucose fluctuation amplitude (MAGE), coefficient of variation (CV), mean absolute difference of daytime glucose (MODD), mean daily risk range (ADDR), and M value. TIR was assessed as the percentage of time of glucose in the 3.9-10 mmol/L range over a 24-h period. Time above range (TAR) represented the percentage of time blood sugar is above the level of 10 mmol/L.

The subjects were wearing thin clothing, supine, resting for more than 5 min before measurement, and ABI was automatically measured using the Omron Arteriosclerosis detector (BP-203RPEII, Japan). Blood pressure was measured in all extremities during the same cardiac cycle and left and right sides’ ABI were displayed. Based on the criteria recommended by the American Heart Association [17] and confirmed in the 2003 Consensus statement of the American Diabetes Association [9], ABI ≤ 0.9 was determined as the threshold for PAD diagnosis, and ABI ≥ 1.3 as the threshold for the presence of calcification. Patients were grouped into one of three ABI subcategories: The ABI of both lower extremities > 0.9 and < 1.3 was "normal", the ABI of at least one lower extremity ≤ 0.9 was "Low", and the ABI of at least one lower extremity ≥ 1.3 and the ABI of the other lower extremity > 0.9 was "High". Both "Low" and "High" were classified as "Abnormal ABI".

### Statistical analyses

SPSS 26.0 software was applied for statistical analysis. Continuous data with normal distribution were expressed in mean ± SD, and inter-group comparison was performed by one-way ANOVA. Abnormal distribution measurement data were expressed as medians (lower and upper quartiles) [M (QL, QU)], and variables among multiple groups were compared by the non-parametric Kruskal–Wallis test. The categorical variables were expressed as count (percentages) [N (%)] and the χ2 test for which was used. Spearman analysis was used to analyze the correlation between abnormal ABI and other indexes. The independent factors contributing to abnormal ABI were analyzed by logistic regression analysis. P < 0. 05 was of statistically significant difference.

## Results

### The comparison of clinical characteristics among each group of ABI

The comparisons of cardiovascular risk factors and other descriptive indicators of all individuals were exhibited in Table 1. The median (upper and lower quartile) age of all subjects was 55.0 (47.0, 64.5) years, and the median (upper and lower quartile) diabetes duration was 7.0 (2.0, 13.5) years. There were 323 patients with normal ABI, and 82 patients with abnormal ABI, including low ABI (n = 20) and high ABI (n = 62), with a prevalence of 4.9% and 15.3%, respectively. Among the three groups, the differences in hypertension prevalence rate, diabetic retinopathy prevalence rate, age, diabetes duration, UREA, Scr, ACR, and LDL were statistically significant (P < 0.05). Table 2 shows the data of variables associated with glycemic control. Among the three groups, the differences in TIR, TAR, and MBG were statistically significant (P < 0.05). The median TIR values (upper and lower quartile) were 67.77 (46.05, 83.93), 74.00 (56.00, 86.15), and 61.17 (42.15, 80.99) in the low, normal, and high ABI groups, respectively.

**Table 1:** Clinical characteristics of low, normal, and high ABI groups

	Total	Low ABI	Normal ABI	High ABI	χ^2^/t/z	P
N	405	20	323	62		
Male (n, %)	278, 68.6%	14, 70.0%	220, 68.1%	44, 71.0%	0.215	0.898
**Age (y)**	**55.0 (47.0,64.5)**	**59.5 (54.0,65.0)**	**55.0 (47.0,63.0)**	**60.0 (51.0,68.0)**	**6.973**	**0.031**
**Diabetes duration (y)**	**7.0 (2.0,13.5)**	**13.5 (4.5,20.0)**	**6.0 (2.0,12.0)**	**10.0 (3.0,15.3)**	**11.866**	**0.003**
BMI	24.80 (22.70,27.30)	25.75 (23.08,27.08)	24.70 (22.50,27.40)	25.40 (23.40,27.33)	1.253	0.534
**Hypertension (n, %)**	**214, 52.8%**	**14, 70.0%**	**161, 49.8%**	**39, 62.9%**	**6.045**	**0.049**
Smoking (n, %)	115, 28.4%	9, 50.0%	89, 29.8%	17, 30.9%	3.255	0.196
**Diabetic retinopathy (n, %)**	**79, 19.5%**	**2, 10.0%**	**58, 18.1%**	**19, 31.1%**	**6.744**	**0.034**
Diabetic kidney disease (n, %)	59, 14.6%	3, 15.0%	42, 13.0%	14, 22.6%	3.837	0.147
CRP (mg/L)	0.80 (0.50,2.20)	1.10 (0.50,5.53)	0.80 (0.50,2.10)	0.80 (0.50,3.05)	1.619	0.445
**UREA** (**mmol/L**)	**5.70 (4.70,6.70)**	**5.75 (5.18,7.60)**	**5.60 (4.70,6.60)**	**6.20 (5.00,8.00)**	**9.351**	**0.009**
**Scr (μmol/L)**	**58.00 (48.10,71.00)**	**68.80 (51.50,112.50)**	**57.00 (48.00,68.25)**	**59.60 (48.90,76.50)**	**8.596**	**0.014**
UA (μmol/L)	313.00 (255.00,394.00)	329.00 (274.50,426.25)	313.00 (256.50,329.00)	305.50 (250.00,387.25)	1.745	0.418
**ACR**	**11.80 (6.00,53.50)**	**54.40 (5.45,188.38)**	**10.30 (5.90,41.00)**	**22.35 (7.00,264.10)**	**10.618**	**0.005**
TC (mmol/L)	4.39 (3.68,5.09)	4.69 (3.59,5.65)	4.40 (3.67,5.13)	4.27 (3.75,4.65)	2.962	0.227
TG (mmol/L)	1.49 (1.01,2.17)	1.69 (1.06,2.11)	1.49 (1.04,2.21)	1.38 (0.89,2.08)	1.506	0.471
HDL (mmol/L)	1.06 (0.90,1.24)	0.99 (0.84,1.13)	1.06 (0.91,1.25)	1.08 (0.94,1.27)	3.194	0.203
**LDL (mmol/L)**	**2.71 ± 0.90**	**3.11 ± 1.00**	**2.73 ± 0.89**	**2.50 ± 0.82**	**3.623**	**0.028**
HbA1C (%)	8.70 (7.10,10.10)	8.30 (6.88,9.98)	8.70 (7.10,10.10)	8.65 (7.10,10.00)	0.288	0.866

^a^BMI: body mass index; CRP: C-reactive protein; Scr: serum creatinine; UA: blood uric acid; ACR: urinary albumin/creatinine ratio; TC: total cholesterol; TG; triglyceride; HDL: high-density lipoprotein; LDL: low‐density lipoprotein; HbA1C: hemoglobin A1C

^b^Continuous data with normal distribution were expressed in mean ± SD, and inter-group comparison was performed by one-way ANOVA. Abnormal distribution measurement data were expressed as medians (lower and upper quartiles) [M (QL, QU)], and variables among multiple groups were compared by non-parametric Kruskal-Wallis test. The categorical variables were expressed as count (percentages) [N (%)] and χ2 test for which was used

The bolded values in the table highlight results with P value < 0.05

**Table 2:** Clinical characteristics of low, normal, and high ABI groups

	Total	Low ABI	Normal ABI	High ABI	χ^2^/t/z	P
N	405	20	323	62		
**TIR (3.9–10 mmol/L) (%)**	**72.96 (52.28,85.56)**	**67.77 (46.05,83.93)**	**74.00 (56.00,86.15)**	**61.17 (42.15,80.99)**	**9.527**	**0.009**
**TAR% (> 10 mmol/L) (%)**	**26.41 (13.40,46.93)**	**31.93 (15.06,53.95)**	**25.67 (13.08,43.26)**	**36.52 (14.38,57.85)**	**6.750**	**0.034**
**MBG (mmol/L)**	**8.83 (7.88,10.31)**	**9.04 (7.95,10.76)**	**8.76 (7.83,10.04)**	**9.91 (8.23,10.88)**	**6.251**	**0.044**
SD (mmol/L)	2.31 (1.76,2.95)	2.59 (1.59,3.08)	2.31 (1.77,2.94)	2.28 (1.75,3.12)	0.151	0.927
MODD (mmol/L)	2.04 (1.47,2.77)	2.10 (1.48,2.99)	2.04 (1.45,2.76)	2.00 (1.50,3.00)	0.096	0.953
MAGE (mmol/L)	4.48 (3.39,5.59)	3.80 (3.37,5.88)	4.55 (3.39,5.67)	4.38 (3.28,5.15)	0.735	0.693
ADDR (mmol/L)	21.80 (15.31,29.58)	20.24 (14.60,29.19)	21.37 (15.47,29.25)	24.92 (15.18,31.64)	1.446	0.485
M value (mmol/L)	6.64 (3.04,12.22)	7.59 (2.78,13.47)	6.20 (3.01,11.59)	10.10 (3.44,17.32)	5.215	0.074
CV[M (QL, QU)]	0.25 (0.21,0.32)	0.25 (0.18,0.34)	0.26 (0.21,0.32)	0.25 (0.20,0.29)	1.229	0.541

^a^TIR: time in range; TAR: time above range; MBG: mean blood glucose; SD: standard deviation; MODD: mean of daily differences; MAGE: mean amplitude of glucose excursions; ADDR: average daily risk range; CV: coefficient of variation

^b^Continuous data with normal distribution were expressed in mean ± SD, and inter-group comparison was performed by one-way ANOVA. Abnormal distribution measurement data were expressed as medians (lower and upper quartiles) [M (QL, QU)], and variables among multiple groups were compared by non-parametric Kruskal-Wallis test. The categorical variables were expressed as count (percentages) [N (%)] and χ2 test for which was used

The bolded values in the table highlight results with P value < 0.05

We then divided the ABI into normal and abnormal groups. Compared with the normal ABI group, the abnormal ABI group has significantly increased hypertension, age, diabetes duration, UREA, Scr, ACR, TAR, MBG, and M values, and significantly decreased TIR (P < 0.05). The median TIR (upper and lower quartile) was 74.00 (56.00, 86.15) and 61.87 (43.74, 83.56) in normal and abnormal ABI groups, respectively. However, no significant inter-group differences were found in HbA1C.

### The comparison of clinical characteristics by quartiles of TIR

Patients were stratified according to TIR quartiles ((Q1): ≤ 52.30%; (Q2): 52.30–72.96%; (Q3): 72.96–85.53%; (Q4): > 85.53%), the characteristics were shown in Table 3. Increased age and diabetes duration were associated with decreased TIR. With ascending quartiles of TIR, downtrends were observed in the prevalence rate of diabetic retinopathy, ACR, HbA1C(%), SD, MODD, MAGE, MBG, ADDR, M value, and CV of type 2 diabetes patients(P < 0.05); on the contrary, Male proportion and UREA value showed an elevated trend (P < 0.05).

**Table 3:** Clinical Characteristics of TIR Quartiles (Q1-Q4)

	Q1	Q2	Q3	Q4	χ^2^/z	P
N	102	101	101	101		
**Male (n, %)**	**60, 58.8**	**66, 65.3**	**75, 74.3**	**77, 76.2**	**9.265**	**0.026**
**Age (y)**	**59.0 (49.8,67.3)**	**56.0 (48.5,66.0)**	**53.0 (45.0,61.0)**	**54.0 (44.0,61.0)**	**13.301**	**0.004**
**Diabetes duration (y)**	**10.0 (2.8,16.3)**	**9.0 (4.0,15.0)**	**7.0 (2.0,12.5)**	**5.0 (1.0,10.0)**	**13.398**	**0.004**
BMI	25.20 (22.78,28.10)	24.80 (22.50,26.90)	24.40 (22.90,27.00)	25.20 (22.30,28.15)	2.453	0.484
Hypertension (n, %)	57, 55.9%	53, 52.5%	49, 48.5%	55, 54.5%	1.248	0.741
Smoking (n, %)	25, 27.2%	27, 28.7%	31, 33.0%	32, 34.8%	1.646	0.649
**Diabetic retinopathy (n, %)**	**17, 17.2%**	**32, 31.7%**	**17, 17.0%**	**13, 12.9%**	**13.006**	**0.005**
Diabetic kidney disease (n, %)	14, 13.7%	16, 15.8%	19, 18.8%	10, 9.9%	3.419	0.331
CRP (mg/L)	0.70 (0.50,2.08)	0.80 (0.50,2.30)	0.75 (0.50,1.85)	1.00 (0.50,2.55)	4.526	0.210
UREA (mmol/L)	5.60 (4.60,6.80)	5.60 (4.40,6.70)	5.85 (5.03,6.88)	5.60 (4.85,6.60)	2.845	0.416
Scr (μmol/L)	54.00 (47.00,70.00)	56.00 (46.95,67.25)	60.00 (50.00,72.83)	62.00 (52.00,71.00)	7.143	0.067
**UA (μmol/L)**	**296.00 (226.00,352.00)**	**306.00 (260.00,388.50)**	**314.50 (259.25,390.50)**	**357.00 (280.00,423.50)**	**16.728**	**0.001**
**ACR**	**14.10 (7.60,85.50)**	**12.80 (6.20,72.35)**	**8.90 (5.10,34.80)**	**10.90 (5.40,41.05)**	**9.809**	**0.020**
TC (mmol/L)	4.54 (3.91,5.36)	4.44 (3.74,5.02)	4.38 (3.71,5.06)	4.18 (3.53,5.09)	4.381	0.223
TG (mmol/L)	1.50 (0.98,2.07)	1.49 (0.99,2.16)	1.49 (0.96,2.37)	1.48 (1.07,2.23)	0.321	0.956
HDL (mmol/L)	1.08 (0.92,1.35)	1.05 (0.91,1.25)	1.09 (0.90,1.30)	1.01 (0.89,1.19)	3.430	0.330
LDL (mmol/L)	2.89 ± 0.91	2.71 ± 0.90	2.70 ± 0.92	2.56 ± 0.83	2.282	0.079
**HbA1C (%)**	**9.90 (8.45,10.90)**	**9.00 (8.00,10.35)**	**8.20 (7.10,9.70)**	**7.10 (6.40,8.50)**	**73.749**	** < 0.001**
**MBG (mmol/L)**	**11.03 (10.59,11.72)**	**9.47 (9.09,9.92)**	**8.33 (7.90,8.76)**	**7.28 (6.77,7.84)**	**330.002**	** < 0.001**
**SD (mmol/L)**	**3.00 (2.43,4.03)**	**2.69 (2.27,3.28)**	**2.19 (1.88,2.65)**	**1.51 (1.31,1.79)**	**177.368**	** < 0.001**
**MODD (mmol/L)**	**2.96 (2.22,4.05)**	**2.42 (1.98,3.15)**	**1.82 (1.49,2.34)**	**1.28 (1.01,1.56)**	**172.215**	** < 0.001**
**MAGE (mmol/L)**	**5.22 (4.18,6.55)**	**4.87 (3.85,6.18)**	**4.65 (3.46,5.53)**	**3.39 (2.89,4.12)**	**86.929**	** < 0.001**
**ADDR (mmol/L)**	**32.87 (27.74,39.30)**	**26.88 (21.92,30.37)**	**18.83 (18.83,23.19)**	**11.65 (8.72,15.32)**	**237.058**	** < 0.001**
**M value (mmol/L)**	**17.29 (12.45,25.57)**	**9.13 (7.12,11.69)**	**4.34 (3.59,6.18)**	**1.88 (1.10,2.72)**	**316.832**	** < 0.001**
**CV[M (QL, QU)]**	**0.27 (0.22,0.36)**	**0.29 (0.23,0.35)**	**0.26 (0.22,0.32)**	**0.21 (0.,17, 0.26)**	**55.534**	** < 0.001**

^a^BMI: body mass index; CRP: C-reactive protein; Scr: serum creatinine; UA: blood uric acid; ACR: urinary albumin/creatinine ratio; TC: total cholesterol; TG; triglyceride; HDL: high-density lipoprotein; LDL: low‐density lipoprotein; HbA1C: hemoglobin A1C; MBG: mean blood glucose; SD: standard deviation; MODD: mean of daily differences; MAGE: mean amplitude of glucose excursions; ADDR: average daily risk range; CV: coefficient of variation

^b^Continuous data with normal distribution were expressed in mean ± SD, and inter-group comparison was performed by one-way ANOVA. Abnormal distribution measurement data were expressed as medians (lower and upper quartiles) [M (QL, QU)], and variables among multiple groups were compared by non-parametric Kruskal-Wallis test. The categorical variables were expressed as count (percentages) [N (%)] and χ2 test for which was used

The bolded values in the table highlight results with P value < 0.05

### Prevalence of abnormal ABI in different quartiles (Q1-Q4) of TIR

**Fig. 1:**
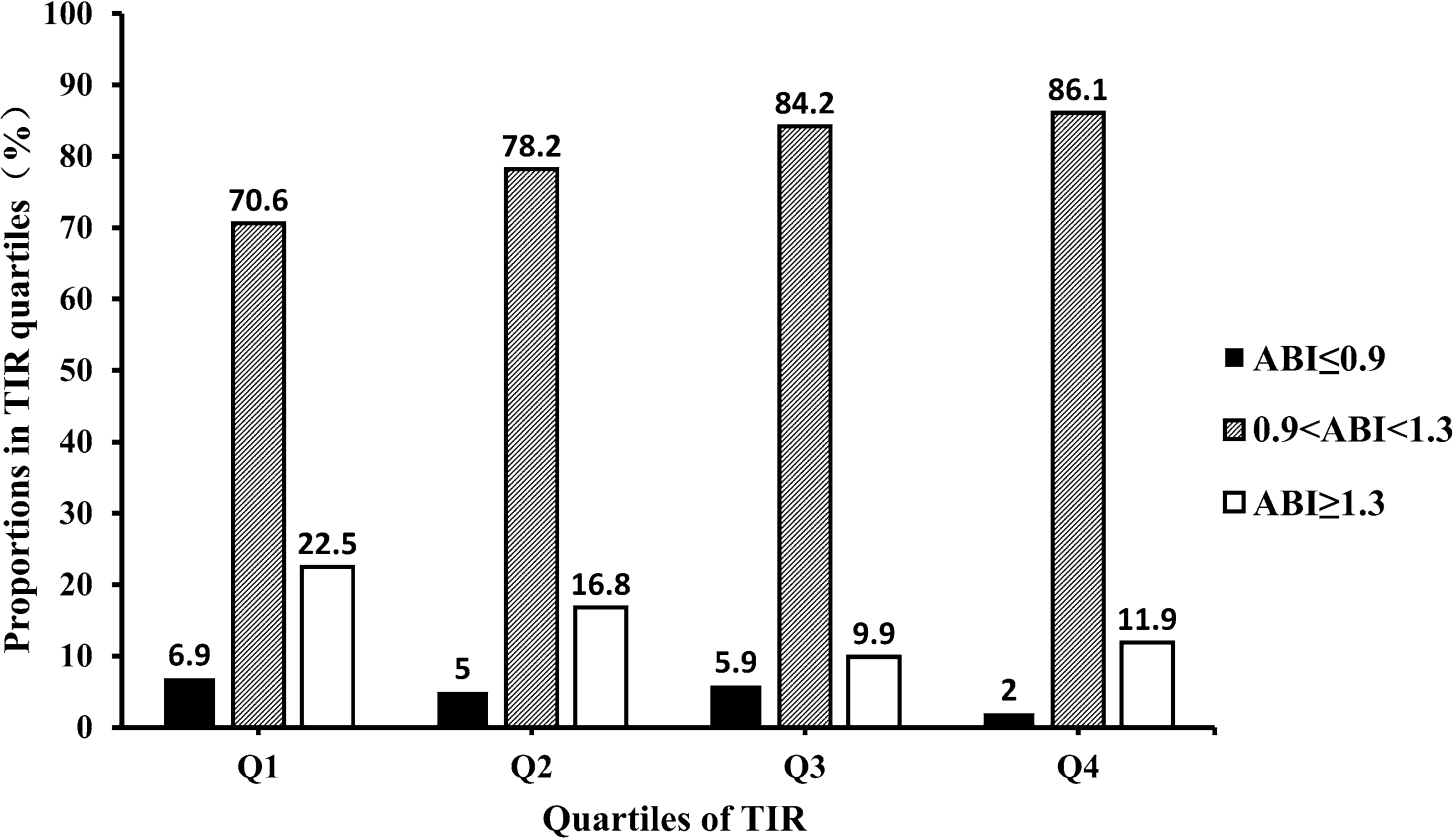
Prevalence of "Abnormally Low ABI" and "Abnormally High ABI" in Different Quartiles of TIR (Q1-Q4) ^a^ABI: ankle-brachial index. ^b^TIR: time in range. (Q1): ≤ 52.30%; (Q2): 52.30-72.96%; (Q3): 72.96-85.53%; (Q4): > 85.53%. ^c^ As shown in this figure, with ascending quartiles of TIR, the percentage of both low ABI and high ABI displayed downward trends(P = 0.084). P value for the significant difference among the groups was determined by Fisher's exact test.

As shown in Table 4, with ascending quartiles of TIR, the prevalence rate of both low ABI and high ABI displayed downward trends. The number and proportion of abnormal ABI in TIR Q1, Q2, Q3, and Q4 were 30 (29.4%), 22 (21.8%), 16 (15.8%), and 14 (13.9%) (Fig. 1).

**Table 4:** Number and percentage of patients in the low, high, normal, and abnormal ABI groups based on TIR quartiles (Q1 to Q4)

	Q1 N (%)	Q2 N (%)	Q3 N (%)	Q4 N (%)	Total	P
N	102	101	101	101	405	
0.9 < ABI < 1.3	72 (70.6)	79 (78.2)	85 (84.2)	87 (86.1)	323	0.084^b^
ABI ≤ 0.9	7 (6.9)	5 (5.0)	6 (5.9)	2 (2.0)	20	
ABI ≥ 1.3	23 (22.5)	17 (16.8)	10 (9.9)	12 (11.9)	62	
0.9 < ABI < 1.3	72 (70.6)	79 (78.2)	85 (84.2)	87 (86.1)	323	0.026^c^
ABI ≤ 0.9 or ABI ≥ 1.3	30 (29.4)	22 (21.8)	16 (15.8)	14 (13.9)	82	

^a^TIR: time in range; ABI: ankle-brachial index

^b^P values were calculated using Fisher's exact test.

^c^P values were calculated using Pearson chi-square test

### The correlation of TIR and other parameters with abnormal ABI

Spearman correlation analysis was performed on parameters and abnormal ABI. As shown in Table 5, TIR was negatively correlated with abnormal ABI (P < 0.05), while hypertension, age, diabetes duration, UREA, Scr, ACR, TAR, MBG, and M values were positively correlated with abnormal ABI (P < 0.05).

**Table 5:** Correlation between TIR and various indicators and abnormal ABI values

	Abnormal ABI	
	Spearman's rho	P value
Male (n, %)	−0.023	0.649
**Age (y)**	**0.131**	**0.008**
**Diabetes duration (y)**	**0.168**	**0.001**
BMI	0.055	0.271
**Hypertension (n, %)**	**0.119**	**0.017**
Smoking (n, %)	0.050	0.334
Diabetic retinopathy (n, %)	0.079	0.115
Diabetic kidney disease (n, %)	0.088	0.077
CRP (mg/L)	0.058	0.261
**UREA** (**mmol/L**)	**0.152**	**0.002**
**Scr (μmol/L)**	**0.123**	**0.014**
UA (μmol/L)	0.012	0.805
**ACR**	**0.173**	**0.001**
TC (mmol/L)	−0.047	0.344
TG (mmol/L)	−0.041	0.414
HDL (mmol/L)	−0.019	0.705
LDL (mmol/L)	−0.029	0.574
HbA1C (%)	−0.025	0.623
**TIR (3.9–10 mmol/L) (%)**	−**0.152**	**0.002**
**TAR% (> 10 mmol/L) (%)**	**0.128**	**0.010**
**MBG (mmol/L)**	**0.119**	**0.016**
SD (mmol/L)	0.013	0.792
MODD (mmol/L)	0.016	0.757
MAGE (mmol/L)	−0.041	0.417
ADDR (mmol/L)	0.045	0.381
**M value (mmol/L)**	**0.108**	**0.030**
CV[M (QL, QU)]	−0.055	0.270

^a^BMI: body mass index; CRP: C-reactive protein; Scr: serum creatinine; UA: blood uric acid; ACR: urinary albumin/creatinine ratio; TC: total cholesterol; TG; triglyceride; HDL: high-density lipoprotein; LDL: low‐density lipoprotein; HbA1C: hemoglobin A1C; TIR: time in range; TAR: time above range; MBG: mean blood glucose; SD: standard deviation; MODD: mean of daily differences; MAGE: mean amplitude of glucose excursions; ADDR: average daily risk range; CV: coefficient of variation

The bolded values in the table highlight results with P value < 0.05

### Associations between TIR and abnormal ABI after controlling for confounding factors

Binary logistic regression was used to study the independent correlation between TIR and ABI abnormalities. In the Model 1 for which no confounding factors were adjusted, TIR (odds ratio (OR) 0.979, 95% confidence interval (CI) 0.967–0.990, P < 0.001) was significantly associated with the presence of abnormal ABI values. After adjusting for cardiovascular risk factors and other indicators including age, diabetes duration, sex, blood pressure, lipid profiles, UREA, Scr, ACR, BMI, and HbA1C(%)(model 2), the data revealed that TIR (OR 0.981, 95% CI 0.965–0.997, P < 0.05) were still obviously correlated with the presence of abnormal ABI values. Furthermore, after adjusting for other GV metrics, the association persisted (model 3–8, P < 0.05). However, there was no correlation between HbA1C and abnormal ABI values in any of the models (Table 6).

**Table 6:** Logistic regression analysis of influencing factors of abnormal ABI values

	Abnormal ABI		
	OR	95% CI	P value
Model 1			
** TIR**	**0.979**	**0.967–0.990**	** < 0.001**
Model 2			
** TIR**	**0.981**	**0.965–0.997**	**0.020**
HbA1C	0.876	0.732–1.049	0.150
Model 3			
** TIR**	**0.978**	**0.960–0.996**	**0.016**
HbA1C	0.913	0.757–1.102	0.345
SD	0.842	0.577–1.228	0.371
Model 4			
** TIR**	**0.977**	**0.960–0.995**	**0.013**
HbA1C	0.908	0.752–1.098	0.319
MAGE	0.860	0.715–1.035	0.110
Model 5			
** TIR**	**0.980**	**0.964–0.996**	**0.016**
HbA1C	0.927	0.767–1.119	0.428
CV	0.025	< 0.001–1.763	0.089
Model 6			
** TIR**	**0.972**	**0.949–0.995**	**0.016**
HbA1C	0.895	0.739–1.084	0.256
ADDR	0.968	0.925–1.013	0.161
Model 7			
** TIR**	**0.976**	**0.957–0.995**	**0.015**
HbA1C	0.896	0.735–1.091	0.274
MODD	0.752	0.500–1.132	0.172
Model 8			
** TIR**	**0.975**	**0.953–0.998**	**0.032**
HbA1C	0.900	0.750–1.080	0.256
M value	0.982	0.935–1.032	0.476

^a^TIR: time in range; HbA1C: hemoglobin A1C; SD: standard deviation; MAGE: mean amplitude of glucose excursions; CV: coefficient of variation; ADDR: average daily risk range; MODD: mean of daily differences; OR: odds ratio; CI: confidence interval

^b^Confounding factors were not adjusted in Model 1. Model 2 was adjusted for age, diabetes duration, gender, blood pressure, lipid profiles, Scr, UREA, ACR, BMI, and HbA1C (%). Model 3 was adjusted for variables as in Model 2 and for SD. Model 4 was adjusted for variables as in Model 2 and for MAGE; Model 5 was adjusted for variables as in Model 2 and for CV. Model 6 was adjusted for variables as in Model 2 and for ADDR; Model 7 was adjusted for variables as in Model 2 and for MODD; Model 8 was adjusted for variables as in Model 2 and for M values

The bolded values in the table highlight results with P value < 0.05

## Discussion

Diabetes as well as its complications has become increasingly serious public health burden. Peripheral vascular disease, heart disease, and stroke are all highly prevalent in diabetes patients, and traditional microvascular complications such as retinopathy, nephropathy, and neuropathy also occur frequently [1]. ABI was proposed by Winsor et al. in 1950 and was initially used for the non-invasive diagnosis of lower limb PAD, which is a kind of diffuse atherosclerotic vascular disease and has a prevalence ranging from 21.1% to 38.5% in > 30 year-old diabetic patients [18–20], increasing with age and the presence of cardiovascular risk factors [8]. Studies have shown that diabetes is the main risk factor for the onset of PAD [21]. Later, ABI has become an indicator of vascular atherosclerosis, as well as a prognostic indicator of cardiovascular events and dysfunction [6]. Low ABI is a major risk factor for cardiovascular morbidity and mortality in diabetic patients [22, 23], and higher-than-normal ABI also significantly predicts subsequent all-cause mortality [24]. Multiple studies have confirmed that abnormal ABI is associated with an increased risk of cardiovascular or all-cause mortality and major adverse cardiovascular events in diabetes patients. Subgroup analysis showed that abnormally low and high ABI had similar values in predicting cardiovascular mortality [25] and there was a U-shaped association between ABI values and cardiovascular mortality [26]. Although PAD is common in diabetics, it’s still not fully recognized, and its diagnosis is often difficult when patients have peripheral neuropathy, as the latter condition can mask pain [8]. Therefore, defining the etiology, investigating risk factors, and exploring possible early detection and treatment strategies are essential for PAD prevention and control.

Numerous studies have shown significant clinical benefits of using CGM in patients with diabetes, regardless of insulin administration [27]. CGM provides a full range of glucose parameters including TIR, which fills the blind spot of HbA1C and traditional SMBG monitoring and is one of the future trends of diabetes metabolic monitoring. As a new indicator, TIR is a simple and intuitive metric that provides information about the quality of glucose control, reporting the time at which blood sugar levels reached a given target during a given period, allowing personalized analysis, and taking into account individual factors such as diabetes type, age, pregnancy status, and complications. A recent study of 4268 patients found that optimal TIR was the greatest motivator for patients with type 1 diabetes to voluntarily choose a treatment [28]. Another online survey of 3455 patients with diabetes found that TIR was the primary indicator of most concern in patients with type 1 diabetes and type 2 diabetes with or without insulin therapy. In addition, TIR better reflected the effect of acute glucose intervention than HbA1c alone [29], suggesting its possible higher value than HbA1C in evaluating treatment options. The 2017 International Consensus on Use of Continuous Glucose Monitoring recommended TIR as one of the 14 key indicators to be included in the CGM standard report [30]. In 2019, the ATTD panel updated its recommendations in the International Consensus on TIR, positioning TIR as one of the most important indicators in diabetes management and reaching a consensus on glycemic cutoff points and target timing for personalization in different groups of patients [27]. In summary, TIR has become a key indicator for assessing glucose control, a simple and intuitive reflection of the acute effects of treatment and lifestyle changes, and a convenient tool to help patients better understand and manage their condition.

In clinical trials, TIR has been shown to be negatively associated with HbA1c and associated with the risk of long-term diabetic complications as an endpoint. Regarding diabetic microvascular complications, relevant studies have confirmed that TIR has significantly negative correlations with the risk of all stages of diabetic retinopathy, microalbuminuria outcomes [31, 32], proteinuria [33], and diabetic peripheral neuropathy [34–36]. As for macrovascular complications of diabetes, TIR is significantly negatively associated with the risk of abnormal carotid artery intima-media thickness [37] and long-term all-cause and cardiovascular mortality [38]. In our study, with the increase of TIR, the prevalence rate of diabetic retinopathy, ACR, HbA1C(%), SD, MODD, MAGE, MBG, ADDR, M value, and CV showed a decreasing trend (P < 0.05) in type 2 diabetes patients, which was consistent with previous studies.

The purpose of this study was to explore the relationship between TIR and ABI anomalies. The level of TIR in the abnormal ABI group was lower than that in the normal group (P < 0.05). With the increase of TIR quartiles, the prevalence rate of ABI abnormalities decreased significantly at P < 0.05. Spearman correlation analysis showed that TIR was negatively correlated with ABI (P = 0.002). In logistic regression, TIR (OR: 0.979, 95% CI: 0.967–0.990, P < 0.001) was significantly associated with the presence of abnormal ABI in the unadjusted model. The correlation persisted after adjustment for cardiovascular risk factors and other indicators including age, diabetes duration, sex, blood pressure, lipid profiles, UREA, Scr, ACR, BMI, HbA1C(%), and GV indicators (P < 0.05). Based on our results, we found a significant association between TIR and ABI abnormalities independent of HbA1C. More importantly, lower TIR was associated with the presence of abnormal ABI even after adjusting for GV indicators and cardiovascular risk factors, suggesting that the value of TIR in predicting the risk of ABI abnormalities was independent of GV indicators and other cardiovascular risk factors. The significant association of TIR with ABI revealed a potential association between TIR and PAD and arterial calcification, further supporting TIR as a valuable glucose indicator and a reasonable clinical outcome in scientific research.

As a classical glucose monitoring indicator, HbA1C has been widely used in clinical and scientific research worldwide since the 1990s. Its correlation with TIR has been well studied, and it was estimated that there is a 0.8% (9 mmol/mol) change in HbA1C for every 10% change in TIR [15, 32], which was also confirmed in our study. The association between HbA1C and ABI has also been explored. Some cross-sectional observational studies showed that HbA1C and ABI are correlated, with high HbA1C being an independent risk factor of low ABI (ABI ≤ 0.9) [39]; and patients with HbA1C ≥ 7% had 2.9 times the risk of microalbuminuria ( +) and ABI ≤ 0.90 compared with patients with HbA1C < 7% (P = 0.043 and 0.048, respectively) [40]. However, some studies have come to different conclusions. Studies conducted by Papazafiropoulou et al. [41] and Sayilan et al. [42] showed no correlation between ABI value and HbA1C in type 2 diabetes patients, and Zhengliang et al. showed no correlation between ABI and HbA1C in the Shanghai elderly population (including the diabetic group and control group) [43]. In our study, HbA1C also failed to indicate ABI abnormalities. There was no significant difference in HbA1C between different ABI groups, and the correlation between HbA1C and abnormal ABI was not statistically significant (P = 0.623). Binary logistic regression showed that there was no correlation between HbA1C and abnormal ABI in all models. We suggest that TIR may be a more valuable clinical indicator than HbA1C in indicating ABI abnormalities, but further studies are needed to confirm this conclusion. One possible explanation is that HbA1C is primarily associated with microvascular diseases, while ABI is primarily associated with macrovascular complications such as myocardial infarction, stroke, or PAD [44]. On the other hand, HbA1C cannot accurately reflect hypoglycemia and blood sugar fluctuations, which may also be risk factors for diabetic vascular diseases [45–47]. Therefore, TIR has an advantage over HbA1C in assessing the risk of ABI anomalies.

In fact, for individuals, elevated HbA1C levels do not provide clinicians with specific recommendations for adjusting treatment regimens, and studies have shown considerable inter-individual differences in average blood glucose levels even when patients have normal HbA1C levels. HbA1C is unable to well distinguish the HbA1C components generated by physiological and pathological blood glucose exposure and fluctuation, and cannot reflect the degree of blood glucose fluctuation. Compared with HbA1C, TIR has certain advantages. Firstly, TIR is influenced by all factors affecting daily glucose patterns, and it collects all glucose level data within a given time range and can reflect glucose fluctuations. When HbA1C levels do not reflect hypoglycemia, it may result in false good HbA1C levels in such circumstances. Secondly, TIR is more accessible and intuitive, enabling patients to know about their blood glucose control level more clearly, encouraging patients to control their diabetes, and helping them improve their blood glucose control with real-time data. Thirdly, TIR is a more accurate assessment of glycemic control than HbA1C when HbA1C is inconsistent with mean glucose, in conditions such as iron deficiency or other anemia, hemoglobulin disease, and pregnancy [48]. Some clinical researchers believe that with the accumulation of more evidences and the advancement of CGM research and development, TIR is expected to surpass HbA1C in the future and become one of the main evaluation indicators for blood glucose control and management [49].

According to our results, with the decrease of TIR, SD, MODD, MAGE, MBG, ADDR, M value, and CV of type 2 diabetes patients increased (P < 0.05), that is, the glycemic variability as well as hypoglycemia and hyperglycemia events increased. Some studies have suggested that vascular endothelial dysfunction is considered to be the key pathogenic basis of type 2 diabetes macrovascular complications, and hyperglycemia may cause vascular endothelial dysfunction and accelerate the occurrence of diabetic macrovascular complications [45]. On the side, glycemic variability exacerbates oxidative stress in type 2 diabetes patients, further damages endothelial cells, and leads to the occurrence of diabetic macrovascular complications through increased inflammation or epigenetic changes [46, 47]. These may partly explain why TIR is associated with the macrovascular complications suggested by ABI abnormalities, but the specific mechanisms need to be further investigated.

Previous studies have shown a J-shaped association between the ABI and diabetic mortality and the occurrence of vascular complications, with the risk increasing in the low ABI population and continuing to increase as the ABI decreases [50], but studies on high ABI are still scarce. As for the abnormal changes of ABI values of both low and high in the same state of low TIR, we speculate that there may be the following reasons. Firstly, glucose control level represented by TIR is only one of the main influencing factors for PAD and arterial calcification, and the two diseases are also influenced by various other factors, such as advanced age, diabetes duration, smoking, hypertension, hyperlipidemia, etc. And even if they share the same influencing factors, the contribution to the disease of these factors may be different. In our study, two groups were compared on the premise that there were significant differences among the three ABI groups. On the premise that there were no significant differences among other groups in the same index, only LDL values in the low and high ABI groups showed significant difference (P = 0.009), and LDL in the low ABI group was significantly higher than that in the high ABI group, suggesting that the degree of LDL-mediated atherosclerosis is the most important contributing factor of low ABI, and preliminary indicating that type 2 diabetes patients with higher LDL are more prone to have lower ABI. There was a significant difference in Scr between normal and low ABI groups (P = 0.007), and there were significant differences in TIR (P = 0.003), age (P = 0.023), UREA (P = 0.006), ACR (P = 0.004), TAR (P = 0.015), and MBG (P = 0.014) between normal and high ABI groups, and in diabetes duration between both normal and low (P = 0.023) and normal and high (P = 0.006) ABI groups. We can infer that elevated Scr may be a risk factor for low ABI (consistent with previous studies [5]); advanced age, high UREA, high ACR, and hyperglycemia are more likely to be risk factors for high ABI; and long diabetes duration may be a risk factor for both.

Low ABI has been shown to be associated with many cardiovascular risk factors. Previous studies have shown that the most serious risk factors for PAD were diabetes and smoking; others included advanced age, hypertension, and hyperlipidemia; potential risk factors included CRP, fibrinogen, homocysteine, apolipoprotein B, lipoprotein (a), and elevated plasma viscosity. In multivariate analyses of most studies, TC and low HDL levels were associated with PAD. Genetics, poverty, environmental pollution, and physical inactivity have also been linked to PAD [51]. In diabetics, the risk of PAD increased with age, diabetes duration, insulin use, and the presence of peripheral neuropathy [9]; every 1% increase in HbA1C was associated with a 26–28% increase in the risk of developing PAD, according to the UK Prospective Diabetes Study (UKPDS) [51, 52]. Eraso et al. carried out a study on PAD and prevalence and cumulative risk factor spectrum analysis, and Joosten et al. carried out a study on the relationship between conventional cardiovascular risk factors and male PAD risk factors, and both found that the impact of risk factors is cumulative, and the more the number of risk factors, the greater the risk of PAD [24, 53].

On the other side, studies have shown that high ABI is directly associated with male sex, diabetes, hypertension, BMI, and age, but negatively associated with smoking and hyperlipidemia, with diabetes being the strongest risk factor [6, 54]. Patients with clinical neuropathy, nephropathy, and long diabetes duration were considered to be at high risk for arterial calcification [8]. Genome-Wide Association Study has identified multiple contributing sites associated with atherosclerosis, diabetes, and coronary artery calcification; high glucose levels were observed to be highly associated with vascular calcification and vascular disease [55]. Risk factors for vascular and valve calcification included aging, metabolic syndrome, smoking, and male sex [56]. In addition, according to the pathophysiological process of vascular calcification, its driving factors included elevated serum phosphate, advanced glycation end products, bone morphogenetic protein, inflammatory cytokines, and leptin. Magnesium, antioxidants, vitamin K, and sufficient but not excessive vitamin D status seemed to prevent arterial calcification [57].

In summary, different risk factors for low and high ABI suggest that the pathogenesis of PAD and arterial calcification may differ. And differences in study populations may explain differences between study results.

On the other hand, arterial calcification may cover PAD and lead to a relatively abnormally high ABI. Although the two diseases often coexist, when vascular calcification is present, the ABI cannot detect stenosis due to the decreased compressibility of the arteries [6]. High ABI values are associated with atherosclerosis secondary to arterial calcification and may lead to an underestimation of the prevalence of PAD in diabetes in cases where complex or long-term diabetes leads to more arterial calcification. In fact, among diabetics with high cardiovascular risk and neuropathy and with normal ABI between 0.9 and 1.3, the prevalence of PAD as measured by DUS was 57% [58]; other authors have also reported a high prevalence of PAD in diabetic patients with elevated ABI, estimated to be between 58 and 84% [59, 60]. Researchers suggested that reduced blood flow to the lower extremities in diabetics due to arteriosclerosis may explain the association between high ABI and PAD [61]. In such cases, it is recommended that a duplex ultrasound must be performed to confirm and assess PAD [8].

There are some limitations of our study. Firstly, it was a single-center study with a relatively small sample size, which may lead to limited statistical certainty. Secondly, studies showed that the CGM of 70% of patients in the last 14 days was closely related to 3-month mean glucose, TIR, and hyperglycemia. Our study conducted CGM for only 72 h, which may not be so sufficient [48]. Thirdly, as a cross-sectional study, our study only described the correlation between TIR and ABI abnormalities, but could not provide more information about the causal relationship. Fourthly, single rather than dynamic measurement of the ABI may lead to individual selection bias. Finally, arterial occlusion or calcification had not been confirmed by diagnostic imaging, such as angiography or ultrasound, and computed tomography. In these patients, ABI values may tend to be pseudo-normalized, leading to the misclassification of ABI categories.

## Conclusions

In this cross-sectional study, we have demonstrated that there was a significant association between low TIR and abnormal ABI values in patients with type 2 diabetes, even after adjusting for cardiovascular risk factors and HbA1C, which is the traditional gold standard of glucose control. This suggests that ABI screening for patients with low TIR values should be emphasized in clinical work to identify high-risk patients for early intervention to improve their clinical outcomes. Whether optimized TIR may help reduce the risk of abnormal ABI and further prevent the progression of cardiovascular disease and decrease related mortality in type 2 diabetes patients remains to be further proven by prospective studies.

## Data Availability

The datasets used and analyzed during the current study are available from the corresponding author on reasonable request.

## Abbreviations

TIR: Time in range
ABI: Ankle-brachial index
GV: Glycemic variability
PAD: Peripheral arterial disease
HbA1C: Hemoglobin A1C
CGM: Continuous glucose monitoring
BMI: Body mass index
TC: Total cholesterol
TG: Triglyceride
HDL: High-density lipoprotein
LDL: Low-density lipoprotein
CRP: C-reactive protein
Scr: Serum creatinine
UA: Blood uric acid
ACR: Urinary albumin/creatinine ratio
MBG: Mean blood glucose
SD: Standard deviation
MAGE: Mean glucose fluctuation amplitude
CV: Coefficient of variation
MODD: Mean absolute difference of daytime glucose
ADDR: Mean daily risk range
TAR: Time above range
OR: Odds ratio
CI: Confidence interval
